# Metasurface Lossless-Regulation Mechanism of Dynamic Acoustic Mass for Low-Frequency Aerodynamic Noise Control

**DOI:** 10.3390/ma18225095

**Published:** 2025-11-10

**Authors:** Min Li, Jiuhui Wu

**Affiliations:** 1 School of Mechanical Engineering, Xi’an Jiaotong University, Xi’an 710049, China; 2 State Key Laboratory for Strength and Vibration of Mechanical Structures, Xi’an Jiaotong University, Xi’an 710049, China

**Keywords:** acoustic metasurface, low-frequency aerodynamic noise reduction, lossless-regulation of dynamic acoustic mass, embedded-neck Helmholtz resonator, wind tunnel experiment

## Abstract

To solve the problem of low-frequency aerodynamic noise control of Helmholtz resonators (HR) due to the frequency shift and amplitude reduction in acoustic attenuation caused by increasing fluid flow, the lossless regulation mechanism of the dynamic acoustic mass of a novel embedded-neck Helmholtz resonator (ENHR) metasurface is revealed through finite element simulation and wind tunnel experiments. Firstly, the flow–acoustic coupling aerodynamic simulation model based on the ducted silencer system is established. Then, the physical mechanism of lossless regulation of the dynamic acoustic mass for low-frequency aerodynamic noise reduction under incident fluid flow is studied specifically. Finally, a sub-wavelength and larger broadband ENHR metasurface comprising ten parallel cells is designed, in which an average transmission loss (TL) of 18.7 dB within 70–200 Hz with a Mach number (Ma) of 0.05 is achieved. The lossless regulation mechanism of dynamic acoustic mass with metasurface design would have an extensive potential application value in controlling low-frequency aerodynamic noise.

## 1. Introduction

Aerodynamic noise control has become a hot problem in the fields of aviation, aerospace, navigation, and high-speed ground transportation, the root of which lies in the suppression of fluctuating acoustic pressure [1,2], and the key problems lie in the low-frequency, broadband, and near-zero suppression; thus, the efficient control of low-frequency aerodynamic noise of HR will be the main focus in this study. As the most widely used resonant structure for sound absorption and noise reduction, the aerodynamic noise reduction in HR has attracted much attention. Under incident fluid flow, the flow instability of the shear layer at the HR interface is the key cause of the fluctuating disturbance, and the development trend of the shear layer depends not only on the characteristics of fluid flow but also on its structural characteristics. Large numbers of studies by Comming et al. have demonstrated that the acoustic attenuation of HR decreases proportionally with the increase in flow velocity, which is manifested as an increase in the fundamental resonance frequency and acoustic impedance, as well as a reduction in the transmission loss [3,4,5,6,7,8]. The higher the velocity of fluid flow, the more difficult it becomes to achieve low-frequency aerodynamic noise reduction with HR. Panton et al. investigated, through experimental research, that the geometric shape of the HR orifice has an important influence on the growth of vortexes in the shear layer [9]. Numerical simulation by Ji et al. shows that the Mach number of bias flow and the geometric parameters of orifice have a great influence on the maximum sound absorption and effective band width [10]. Zhao et al. conducted a study on the transmission loss of HR with circular and rectangular orifices in a cold-flow pipe system with a mean flow, which indicated that the geometric orifice and Mach number of the grazing flow have great influence on the aeroacoustics damping performance of HR [11]. Zhang et al. proposed a space-folded metamaterial muffler with wire meshes at the branch inlet for pipe noise reduction and indicated that aerodynamic noise could be significantly suppressed due to the attenuation of flow velocity and vorticity in the branch inlet area caused by the interference of wire meshes on the flow field through simulation and experimental studies [12]. Guan et al. increased the peak value of secondary transmission loss by 2–5 dB and the primary resonance frequency by 20% through changing the neck of HR from cylinder to arc [13]. Zhang et al. adjusted the damping performance of HR by changing the neck area, which significantly improved the stability of the combustion system and reduced the sound pressure level by more than 50 dB [14]. Juan et al. found that the acoustic response with bias flows was particularly sensitive to upstream geometric modification of short circular holes and the adjustment of positive and negative acoustic absorption efficiency could be achieved by designing the upstream chamfer [15]. In our previous studies, a stepped-hole HR metasurface was proposed and a novel zero-impedance matching mechanism for efficient aerodynamic noise reduction is revealed by regulating the phase matching of the incident and scattering field, which makes the wall acoustic pressure near zero and thus inhibits the fluctuating pressure source [16,17,18]. The above studies indicate the quantitative impact of fluid flow on the acoustic attenuation of HR and feasibility of aerodynamic noise control by designing structure characteristics. However, the new mechanism of HR for low-frequency aerodynamic noise reduction should still be further revealed.

In this work, a novel ENHR metasurface with a lossless regulation mechanism of dynamic acoustic mass is proposed aiming at low-frequency and broadband aerodynamic noise reduction. By deeply revealing this mechanism, the influence of structural parameters and flow velocities on the aerodynamic noise reduction performance of ENHR is analyzed. A sub-wavelength multi-cell, parallel-coupled acoustic metasurface is designed to effectively reduce the aerodynamic noise ranging from 70 Hz to 200 Hz when the Mach number of incident flow is 0.05. The paper is organized as follows: in Section 2, the cell structure of the proposed ENHR metasurface is first introduced and a flow–acoustic coupling simulation model is established. Second, in Section 3, the lossless regulation mechanism of dynamic acoustic mass is revealed through finite element simulation. Then, in Section 4, an ENHR metasurface for controlling low-frequency and broadband aerodynamic noise is realized. Finally, in Section 5, several conclusions are drawn.

## 2. Aerodynamic Simulation Modeling of ENHR

### 2.1. Structure of ENHR

**Figure 1:**
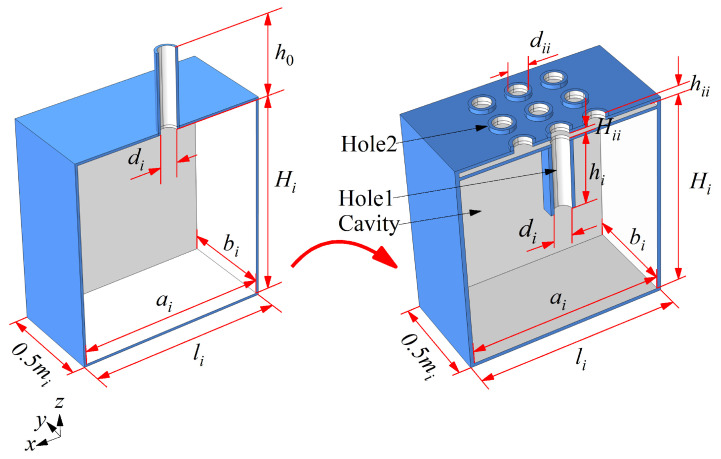
Schematic view of HR and ENHR.

The ENHR is constructed by replacing the neck of HR to an embedded one while keeping other geometric parameters unchanged, as shown in Figure 1, which consists of cylindrical hole 1, with a height of *h*_i_ and diameter of *d_i_*, cylindrical hole 2, with a height of *h_ii_* and diameter of *d_ii_*, a thin rectangular gap with a length and width of *a_i_* and *b_i_* and height of *H_ii_*, a square cavity with a side length of *a_i_* and *b_i_* and depth of *H_i_*, a cell period of *m_i_* and *l_i_*, and so on. The neck diameter of HR is equal to that of hole 1, and the neck height of HR is *h*_0_ = *h_i_* + *h_ii_* + *H_ii_*. The definition of the absorption area is *φ_i_* = *nπd_ii_*^2^*/*(4*S_i_*), where *n* represents the number of holes and *S_i_* = *l_i_∙m_i_* can be defined as the incident area of the cell.

### 2.2. Modeling of Aerodynamic Simulation

**Figure 2:**
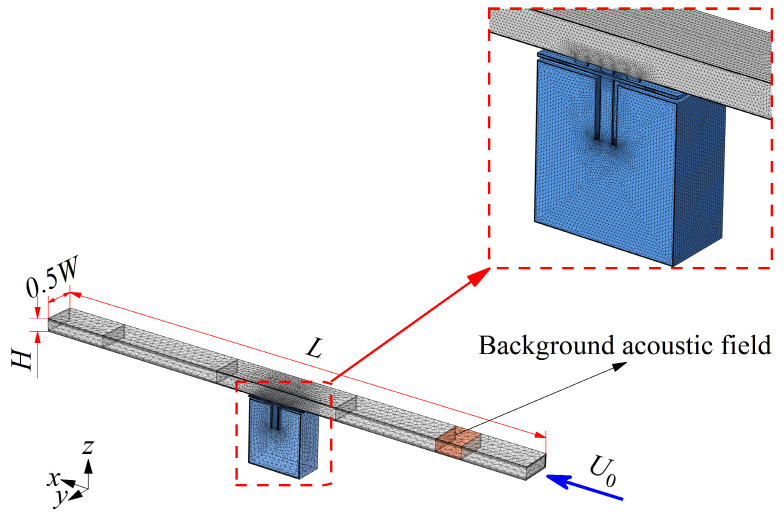
Modeling and mesh division of aerodynamic simulation of ENHR.

The flow–acoustic coupling aerodynamic simulation is performed to explore the physical mechanism of ENHR controlling the low-frequency aerodynamic noise based on computational fluid dynamics (CFD) and acoustics. First, a CFD model is established to solve the flow characteristics and the variables such as flow pressure, velocity, and dynamic viscosity can be calculated. As shown in Figure 2, ENHR elements are located along the longitudinal centerline on the underside of the duct, and the length *L*, width *W*, and height *H* of the duct are 1300 mm, 45 mm, and 10 mm, respectively. The airflow with Ma of 0.05 flows downstream from the *x* axis; the Reynolds number calculated based on the Ma and length (*L*) of duct is 1.49 × 10^6^, indicating that this flow is in a turbulent state. The *k*-*ε* turbulence model is employed to analyze the flow characteristics inside the duct, while the wall function is used to describe the flow characteristics near the wall and inside the structure. The front and rear surface of the computational domain are set as the velocity inlet boundary and pressure outlet boundary, the symmetrical surface is set as the symmetric boundary, and the remaining surfaces are set as no-slip boundaries. The CFD mesh division of the entire computing domain mainly adopts hexahedron and tetrahedron with a total number of 788,315. Six boundary layers with a thickness of 0.02 mm for the first layer and each layer increasing at a ratio of 1:1.2 are arranged in the near-wall area, and a smoothing algorithm is used to minimize the gradients of the mesh size. The verification of this aerodynamic simulation method has been accomplished in our previous studies through mesh convergence studies, as well as comparative analyses with the experimental results of Leinhart et al. [16,17,18,19]. Then, the solved flow variables are mapped to the acoustic model according to the equation of $X_{\mathrm{aci}}-X_{i}-\alpha {h_{m}}^{2}\nabla \cdot \left(\nabla X_{\mathrm{aci}}\right)=0$ (where *X_aci_* and *X_i_* represent mapped variables and flow variables, *i* is the number of coupled variables, *α* is the diffusion constant, and *h_m_* is the mesh size) and the mapped pressure and velocity can be calculated, the accuracy of the mapped study can be verified by comparing the turbulent viscosity and axial velocity vector, respectively, calculated by the flow mesh and acoustic mesh [17]. Finally, the coupling of the flow and acoustics is achieved by establishing an acoustic model using linearized Navier–Stokes equations and mapped variables as inputs to solve the acoustic variables. The plane sound wave passes along the *x* axis and is set as a background acoustic feature to a small domain. The background acoustic variables can be defined as $P_{b}=1\mathrm{Pa}\cdot \exp\left(-ik_{0}x\right),k_{0}=\frac{\omega }{c_{\omega }+U_{0}}$, $T_{b}=\frac{\alpha_{P}T_{0}}{\rho_{\omega }C_{p}}P_{b}$, and $U_{b}=-\frac{1}{i\omega \rho_{\omega }}\frac{\partial P_{b}}{\partial x}$, in which *U_b_*, *P_b_*, and *T_b_*, respectively, represent the background acoustic velocity along the *x* axis, pressure, and temperature, *k*_0_ is the wave number, *c_ω_* is the speed of sound, *ω* is the angular frequency, *ρ_ω_* is the density of air, *T*_0_ is the equilibrium temperature, *α_P_* is the thermal expansion coefficient, and *C_p_* is the heat capacity under constant pressure [16]. In our previous work, the verification of the flow–acoustic coupling simulation of the ducted silencer system for the TL at Mach numbers of 0, 0.05, and 0.1 similar to that of the research by Lienhart et al., was conducted [17,19]. The results show that the peak of TL significantly decreases and shifts to higher frequencies as the Mach number of the fluid flow increases from 0 to 0.1. That is, the fluid flow weakens the attenuation effect of HR on the acoustic field, and this flow–acoustic coupling model can capture well the attenuation process when the acoustic field interacts with turbulence. The simulated TL at different Mach numbers [17] are almost consistent with the test results in reference [19], which further proves that the flow–acoustic coupling model could achieve efficient and accurate simulation of acoustic properties under the influence of fluid flow.

## 3. Lossless Regulation Mechanism of Dynamic Acoustic Mass

**Figure 3:**
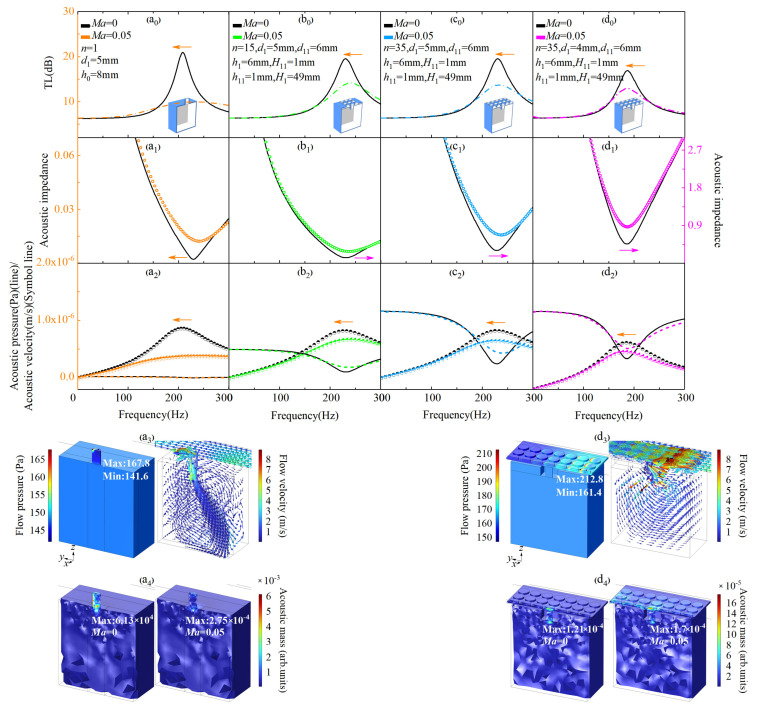
Lossless regulation mechanism of acoustic mass for aerodynamic noise reduction. (**a_0_**–**d_0_**) The TL of HR and ENHR with different parameters when Ma is 0 and 0.05. (**a_1_**–**d_1_**) The acoustic impedance of HR and ENHR with different parameters when Ma is 0 and 0.05. (**a_2_**–**d_2_**) The acoustic pressure and acoustic velocity of HR and ENHR with different parameters shen Ma is 0 and 0.05. (**a_3_**) The flow pressure contour map and the flow velocity vector arrows of HR when Ma is 0.05. (**a_4_**) The acoustic mass of HR when Ma is 0 and 0.05. (**d_3_**) The flow pressure contour map and the flow velocity vector arrows of ENHR when Ma is 0.05. (**d_4_**) The acoustic mass of ENHR when Ma is 0 and 0.05. Arrows of different colors point to the corresponding vertical axis of this figure.

In Figure 3, the flow and acoustic characteristics of ENHR and HR under the incident fluid flow of Ma = 0 and Ma = 0.05 are, respectively, simulated. As shown in Figure 3(a_0_,a_1_), the parameters of HR are *a*_1_ = 45 mm, *b*_1_ = 45 mm, *l*_1_ = 47 mm, *m*_1_ = 47 mm, *H*_1_ = 49 mm, *d*_1_ = 5 mm, and *h*_0_ = 8 mm; the amplitude of TL of the HR when Ma is 0.05 decreases by 10.9 dB from 20.9 dB to 10.0 dB compared with that of Ma = 0; the peak of acoustic impedance increases by 0.01 from 0.002 to 0.012; and the frequency of the peak shifts by 26 Hz from 208 Hz to a higher 234 Hz. In Figure 3(a_2_), as Ma increases from 0 to 0.05, a significant decrease in the normal acoustic velocity and little change in the acoustic pressure of HR are the key causes of the great reduction in TL and rapid increase in impedance peak. In Figure 3(a_3_), when fluid with an Ma of 0.05 flows, there is a counterclockwise pressure difference of 26.2 Pa between the maximum pressure (167.8 Pa) and the minimum pressure (141.6 Pa) at the HR interface, resulting in the counterclockwise shear stress and vortex inside the neck; thus, the formation of a slip velocity consistent with the main flow direction at HR interface occurs [18]. Under the effect of the slip effect, the TL decreases and acoustic impedance increases due to the diminished acoustic attenuation resulting from reduced communication between the main flow and HR. The main flow to the inside of HR is weakened, and the normal acoustic velocity of HR decreases, causing a reduction in the effective length of the HR neck observed as shift in the impedance peak to a higher frequency. According to Figure 3(a_4_), the acoustic mass of HR corresponding to the peak frequency of 208 Hz is 6.13 × 10^−4^ when Ma is 0, while the acoustic mass corresponding to 234 Hz with an Ma of 0.05 is 2.75 × 10^−4^; the decrease in the acoustic mass of HR with the increase in main flow velocity is the direct cause of the shift in the impedance peak to higher frequencies.

In Figure 3(b_0_,b_1_), the neck of the HR is transformed into an embedded one and the number of hole 2 of the ENHR is *n* = 15, the specific parameters are *h*_0_ = *h*_1_ + *h*_11_ + *H*_11_ = 8 mm, *d*_11_ = 6 mm, *d*_1_ = 5 mm, *h*_11_ = 1 mm, *H*_11_ = 1 mm, and *h*_1_ = 6 mm, and the remaining parameters are consistent with HR in Figure 3(a_0_), the amplitude of TL of the ENHR when Ma is 0.05 decreases by 5.4 dB from 19.5 dB to 14.1 dB; the peak of acoustic impedance increases by 0.15 from 0.128 to 0.278; the frequency of the peak shifts by 9 Hz from 231 Hz to a higher 240 Hz; and the frequency shift in ENHR is reduced by 65.4% compared with that of HR, which indicates that ENHR could more effectively suppress the frequency shift in acoustic attenuation caused by the increase in flow velocity compared with HR. In Figure 3(b_2_), great reduction in the normal acoustic velocity and little increase in the acoustic pressure as Ma increases from 0 to 0.05 are the key causes of the significantly increased impedance peak and rapidly decreased TL of ENHR compared with HR.

In Figure 3(c_0_–c_2_), the number of hole 2 of the ENHR is transformed from 15 to 35, and other parameters are consistent with those in Figure 3(b_0_–b_2_). In Figure 3(c_0_,c_1_), the amplitude of TL of the ENHR decreases by 5.8 dB from 19.5 dB to 13.7 dB when Ma is 0.05, and the peak of acoustic impedance increases by 0.37 from 0.3 to 0.67; a great reduction in the normal acoustic velocity and significant increase in the acoustic pressure are the main causes of the increase in acoustic impedance and decrease in TL of ENHR compared with HR as the Ma increases from 0 to 0.05 in Figure 3(c_2_). The frequency of the peak shifts by 2 Hz from 231 Hz to a higher 233 Hz as the Ma increases from 0 to 0.05, which is 77.7% and 92.3% less than that of 15-holes ENHR and HR; increasing the number of hole 2 of the ENHR could greatly improve the efficiency of low-frequency aerodynamic noise reduction.

In Figure 3(d_0_,d_1_), the diameter of hole 1 of ENHR is further transformed from 5 mm in Figure 3(c_0_) to 4 mm, and other parameters are consistent with those in Figure 3(c_0_,c_1_). The amplitude of TL of ENHR when Ma is 0.05 decreases by 4 dB from 16.9 dB to 12.9 dB; the frequency of the peak shifts by −1 Hz from 187 Hz to a lower 186 Hz as the Ma increases from 0 to 0.05. As shown in Figure 3(d_3_,d_4_), when fluid with a Mach number of 0.05 flows through the ENHR interface, the maximum pressure (212.8 Pa) and minimum pressure (161.4 Pa) in one hole of the neck form a pressure difference of 51.4 Pa, and multiple stronger pressure differences cause stronger vortexes in the ENHR neck compared with that of HR. On the one hand, stronger vortexes make the flow velocity at the ENHR interface significantly increase, thereby reducing the acoustic impedance. On the other hand, stronger vortexes enhance the influence of fluid flow within the neck of the ENHR on the main flow, thereby increasing the effective length of the ENHR neck. According to Figure 3(d_3_), the acoustic mass of ENHR corresponding to a peak of 187 Hz is 1.21 × 10^−4^ when Ma is 0, while the acoustic mass corresponding to a peak of 186 Hz is 1.7 × 10^−4^ with an Ma of 0.05; the effective acoustic mass of ENHR does not decrease but increases as the flow velocity increases, and thus, the acoustic attenuation shifting to a higher frequency could be suppressed, which can qualitatively explain the significant frequency shift inhibition of ENHR compared to HR when Ma is 0.05. When subjected to the influence of the incident fluid flow, the shift in acoustic attenuation towards higher frequencies due to the increase in the main flow makes it difficult to effectively control low-frequency aerodynamic noise. Lossless regulation of dynamic acoustic mass refers to adjusting the acoustic mass to prevent the acoustic attenuation from shifting towards higher frequencies as there are increases in main flow; this is the physical mechanism by which ENHR could better reduce the low-frequency aerodynamic sound pressure.

## 4. Metasurface for Low-Frequency, Broadband Aerodynamic Noise Control

### 4.1. Multi-Cell Coupled ENHR Metasurface

**Figure 4:**
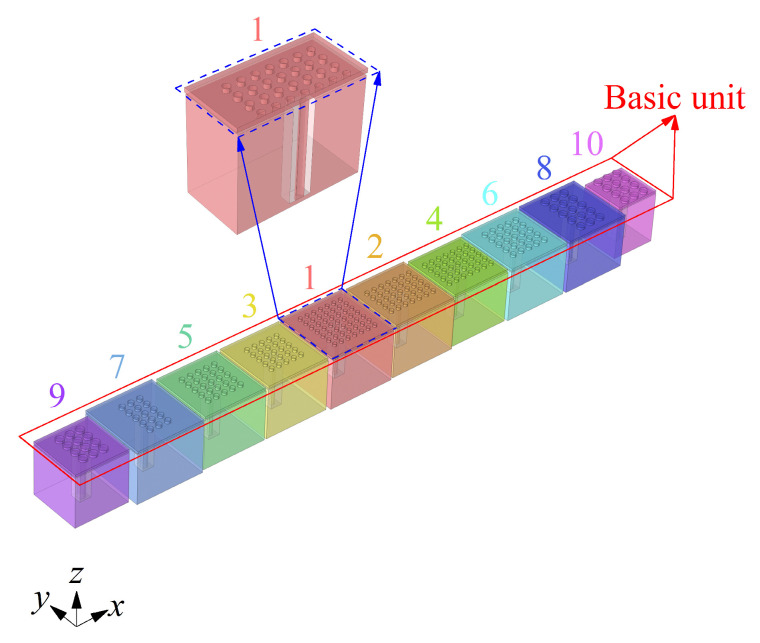
Schematic of basic unit of ENHR metasurface with ten parallel coupled cells.

To realize the broadband reduction in low-frequency aerodynamic noise within 200 Hz of the ducted silencer system, an ENHR metasurface composed of basic noise reduction units formed by ten parallel coupled cells with a thickness of 62 mm is finally constructed based on the lossless regulation mechanism of dynamic acoustic mass, considering the effect of noise distribution characteristics, fluid flow, and key parameters on the aerodynamic performance of ENHR and the limits of the processing accuracy of the sample. The ten cells of the basic unit are numbered sequentially as 1 (red), 2 (orange), 3 (yellow), 4 (yellow-green), 5 (green), 6 (light blue), 7 (blue), 8 (dark blue), 9 (purple), and 10 (rose red), as shown in Figure 4, and the detailed dimensions are shown in Table 1.

### 4.2. Low-Frequency Broadband Aerodynamic Noise Reduction

**Figure 5:**
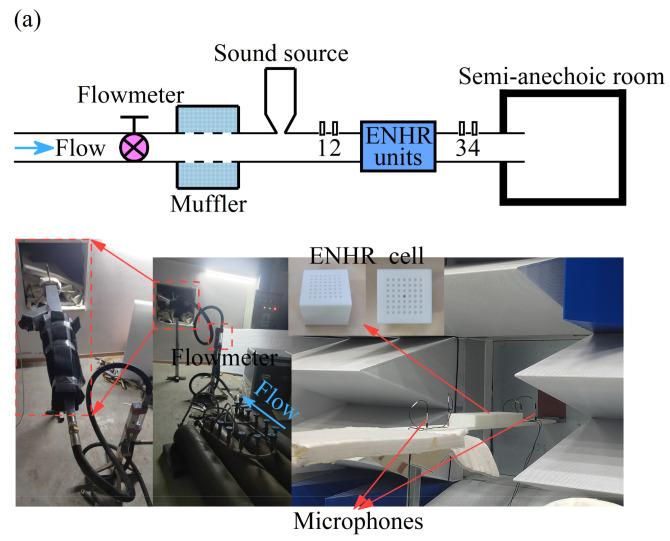

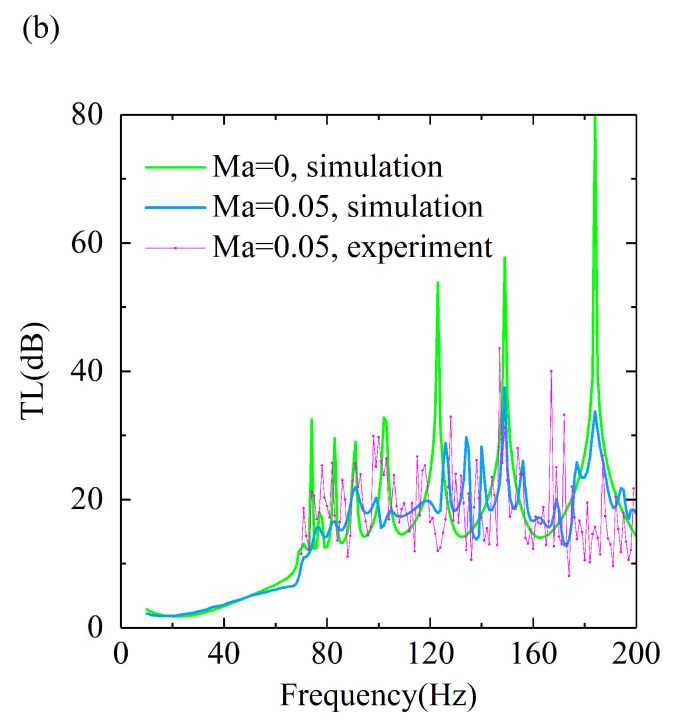
Low-frequency broadband aerodynamic noise control with ENHR metasurface when Ma is 0 and 0.05. (**a**) Installation of experiment. (**b**) Comparison of TL between simulation and experiment.

Figure 5 shows the broadband control of low-frequency aerodynamic noise of the ducted silencer system with an ENHR metasurface containing three groups of basic unit arrays when the Ma is 0 and 0.05, respectively. To validate the results of the theoretical and simulation analysis, ENHR samples are made of photosensitive resin by additive manufacturing, and wind tunnel experiments are conducted as shown in Figure 5a. The airflow during the experiment is maintained consistent with that in simulation at a Ma of 0.05, which is supplied by four nitrogen gas cylinders and could reach a maximum velocity of 20 m/s. The acoustic pressures at the positions of 1, 2, 3, and 4 are measured sequentially using the BK 3050 multi-channel data acquisition instrument and MPA416 1/4-inch microphone, and three repetitive tests are performed for each measuring position to eliminate random errors during the data acquisition. Figure 5b shows the TL of the ducted silencer system with the ENHR metasurface through simulations and experiments when the Ma is, respectively, 0 and 0.05, and a continuous reduction in aerodynamic noise by 18.7 dB in the range of 70~200 Hz is achieved. The measured TL values in the experiment are generally smaller and show the same trend compared to that of the simulated values within the entire frequency range, and the average errors between the simulations and experiments are no more than 5%. These errors can firstly be attributed to the assumption in the simulation that the sound waves propagate in the duct and resonators in the form of plane waves, resulting in the system response presenting a single resonance peak and a smooth TL curve. A series of peaks and valleys appear in the TL curves under coupling and the interference of the fundamental mode and higher-order modes during the experiment, causing significant differences between the peaks of simulations and experiments. Secondly, the rigid assumption for the duct and resonators in the simulation and the occurrence of acoustic–solid coupling in the experiment will result in additional differences in resonance peaks and valleys in the TL curves. The superposition of the TL and random excitation generated by the coupling of sound and vortexes in the experiment and the assumption of uniform flow in the simulation would then lead to larger errors. Furthermore, the ineffectiveness of resonators in noise reduction caused by processing defects such as blockages and the inaccuracy in the control of flow velocity are also important factors for the deviation in simulations and experiments. Finally, the simulations and experiments have demonstrated well the reliability of the simulation method in this study and the effective aerodynamic noise reduction in ENHR metasurfaces ranging from 70 Hz to 200 Hz with a Mach number of 0.05.

## 5. Conclusions

In this study, the lossless regulation mechanism of the dynamic acoustic mass of a sub-wavelength ENHR metasurface composed of a basic unit formed by ten parallel cells for low-frequency aerodynamic noise reduction is presented, and an 18.7 dB of average transmission loss within the range of 70–200 Hz with a Ma of 0.05 is experimented on the basis of the acoustic mass lossless regulation mechanism and multi-order coupled resonance. The realization of the lossless regulation mechanism of dynamic acoustic mass under increasing incident fluid flow can mainly be attributed to the suppression of the frequency shift and increment of acoustic impedance through regulating the acoustic mass, and the vertical acoustic velocity resulted from stronger vortexes generated by a greater shear force and pressure difference in ENHR. Furthermore, adjusting the parameters of ENHR, particularly the number and diameter of holes, could better suppress the flow-induced frequency shift and weakening of acoustic attenuation, thereby more efficiently controlling higher-speed, low-frequency aerodynamic noise. In summary, this ENHR acoustic metasurface based on the dynamic acoustic mass lossless regulation mechanism provides a new approach for controlling aerodynamic noise, especially in the field of low-frequency control.

## Figures and Tables

**Table 1:** Detailed parameters of ENHR cells (unit: mm).

Cell	*a_i_*	*b_i_*	*l_i_*	*n_i_*	*d_i_*	*h_i_*	*H_i_*	*H_ii_*	*n_ii_*	*d_ii_*	*h_ii_*
**1**	59	60	63	1	4	44	45	2	49	3.5	2
**2**	59	58	62	1	5	44	45	2	35	3.9	2
**3**	57	56	59	1	4	44	45	2	25	4	2
**4**	54	54	56	1	4	44	45	2	35	4	2
**5**	57	56	59	1	4	44	45	2	25	4.5	2
**6**	57.5	57	60	1	5	44	45	2	25	5	2
**7**	60	60	63	1	6	44	45	2	15	5	2
**8**	58	58	60	1	7	44	45	2	15	7	2
**9**	48	48	50	1	7	44	45	2	12	7	2
**10**	39.5	39	43	1	7	44	45	2	12	7	2

## Data Availability

The original contributions presented in this study are included in the article. Further inquiries can be directed to the corresponding authors.
